# Supplementary material for: Improving the Aromatic Profiles of Catarratto Wines: Impact of Metschnikowia pulcherrima and Glutathione-Rich Inactivated Yeasts
Source: Antioxidants (Basel). 2023 Feb 10;12(2):439. doi: 10.3390/antiox12020439 (PMC9952006; doi:10.3390/antiox12020439)
Supplement: Supplementary file 1 [file antioxidants-12-00439-s001.zip › antioxidants-2201471-supplementary.pdf]

antioxidants

MDPI

Supplemental Material

Improving the aromatic profiles of Catarratto wines: impact of *Metschnikowia pulcherrima* and glutathione-rich inactivated yeasts

Vincenzo Naselli, Rosario Prestianni, Natale Badalamenti, Michele Matraxia, Antonella Maggio*, Antonio Alfonzo*, Raimondo Gaglio, Paola Vagnoli, Luca Settanni, Maurizio Bruno, Giancarlo Moschetti and Nicola Francesca

Table S1. Chemical parameters determined during alcoholic fermentation time (days): 3, 6, 12, and 18.

Table S2. Chemical parameters determined during steel aging time (month): 1, 3 and 5.

Table S1. Chemical properties determined during alcoholic fermentation time (days): 3, 6, 12, 18.

→ continued

Parameters	Must	Vinification									
		3 days of alcoholic fermentation				S.S.	6 days of alcoholic fermentation				S.S.
		T1	T2	C1	C2		T1	T2	C1	C2	
pH	3.30±0.02	3.15±0.01a	3.10±0.01b	3.14±0.01a	3.06±0.02c	***	3.17±0.02bc	3.16±0.01c	3.21±0.01b	3.27±0.02a	**
TA	5.53±0.09	5.48±0.06b	5.56±0.00ab	5.62±0.04a	5.55±0.02ab	*	5.44±0.10a	5.45±0.05a	5.50±0.06a	5.55±0.07a	n.s.
VA	0.00±0.00	0.00±0.00a	0.00±0.00a	0.00±0.00a	0.00±0.00a	n.s.	0.00±0.00a	0.00±0.00a	0.00±0.00a	0.00±0.00a	n.s.
Rs	223.99±0.92	172.87±1.20a	171.12±1.93a	146.26±0.24b	117.20±0.35c	***	112.89±0.36a	83.19±0.72c	95.75±0.85b	58.79±0.32d	***
Glu	113.05±0.55	79.97±0.19a	78.32±1.16b	60.38±0.21c	61.93±0.24c	***	64.35±0.24a	41.62±0.20b	44.43±0.15c	25.80±0.11d	***
Fru	110.91±0.36	92.90±1.01a	92.80±0.77a	85.88±0.03b	55.27±0.10c	***	48.54±0.12b	41.57±0.52c	51.32±0.70a	32.99±0.21d	***
Eth	0.01±0.00	2.66±0.01c	2.67±0.01c	3.93±0.09b	5.40±0.08a	***	5.69±0.08d	7.16±0.12b	6.57±0.11c	8.44±0.03a	***
Gly	0.92±0.00	2.00±0.03a	2.00±0.01a	2.02±0.02a	2.01±0.01a	n.s.	5.28±0.01a	5.27±0.04a	5.06±0.04b	5.19±0.07a	**
MA	1.90±0.03	1.65±0.02a	1.66±0.02a	1.69±0.02a	1.66±0.03a	n.s.	1.59±0.02a	1.60±0.02a	1.60±0.02a	1.60±0.03a	n.s.
LA	0.00±0.00	0.00±0.00a	0.00±0.00a	0.00±0.00a	0.00±0.00a	n.s.	0.00±0.00a	0.00±0.00a	0.00±0.00a	0.00±0.00a	n.s.
Amm. N	85.42±1.01	161.57±0.31ab	160.86±0.11a	159.51±0.77a	157.27±0.11b	*	146.23±0.28c	147.39±0.31bc	150.96±0.34a	149.91±1.22ab	*
Alpha-AN	33.90±0.39	78.16±0.09c	70.21±0.21d	80.32±0.32b	81.63±0.35a	***	73.44±0.11c	71.39±0.41d	75.97±0.41b	78.66±0.16a	***

Result indicate mean value ± standard deviation of three determinations. Data in the same line followed by the same letter are not significantly different according to Tukey's test. Abbreviations: TA, total titratable acidity (tartaric acid g/l); VA, volatile acidity (acetic acid g/L); RS, reducing sugar (g/L); Glu, glucose (g/L); Fru, fructose (g/L); Eth, ethanol (% v/v); Gly, glycerol (g/L); MA, malic acid (g/L); LA, lactic acid (g/L); Amm. N, ammoniacal nitrogen (mg/L); Alpha-AN, alpha-amino nitrogen (mg/ L); n.d., not determined. P value: *, $P < 0.05$; **, $P < 0.01$; ***, $P < 0.001$; n.s., not significant.

Parameters	Vinification									
	12 days of alcoholic fermentation				S.S.	End of alcoholic fermentation (18 days)				S.S.
	T1	T2	C1	C2		T1	T2	C1	C2	
pH	3.24±0.01c	3.21±0.01c	3.31±0.00b	3.35±0.02a	***	3.41±0.02b	3.43±0.01b	3.47±0.00a	3.51±0.01a	***
TA	5.48±0.03a	5.45±0.07a	5.45±0.07a	5.48±0.08a	n.s.	5.41±0.09a	5.40±0.09a	5.42±0.02a	5.50±0.08a	n.s.
VA	0.00±0.00a	0.00±0.00a	0.00±0.00a	0.00±0.00a	n.s.	0.27±0.00c	0.29±0.00b	0.31±0.00a	0.31±0.00a	***
Rs	49.51±0.34a	28.80±0.12c	47.80±0.20b	27.60±0.30c	***	3.70±0.04a	2.60±0.05d	3.01±0.03c	3.24±0.03b	***
Glu	21.43±0.25a	12.07±0.09b	21.09±0.06a	10.26±0.12c	***	1.10±0.01b	1.10±0.01b	1.62±0.01a	1.11±0.01b	***
Fru	28.08±0.09a	16.73±0.03d	26.71±0.14b	17.34±0.18c	***	2.60±0.03a	1.50±0.04c	1.39±0.02d	2.13±0.02b	***
Eth	9.00±0.11b	10.11±0.06a	9.03±0.03b	10.09±0.08a	***	11.35±0.15a	11.43±0.13a	11.36±0.08a	11.35±0.07a	n.s.
Gly	5.34±0.02b	5.92±0.09a	5.20±0.03b	5.22±0.05b	***	5.58±0.04c	5.31±0.05d	5.85±0.08b	6.57±0.12a	***
MA	1.55±0.01	1.58±0.02	1.47±0.01	1.50±0.01	***	1.49±0.02a	1.50±0.02a	1.36±0.01b	1.28±0.02c	***
LA	0.00±0.00a	0.00±0.00a	0.00±0.00a	0.00±0.00a	n.s.	0.00±0.00a	0.00±0.00a	0.00±0.00a	0.00±0.00a	n.s.
Amm. N	119.88±0.43a	112.28±0.10b	110.38±0.44c	109.22±0.37c	***	49.35±0.27a	48.78±0.17a	45.61±0.21b	44.41±0.13c	***
Alpha-AN	67.32±0.08c	68.74±0.18b	63.84±0.13d	69.79±0.15a	***	55.21±0.08b	53.19±0.06c	56.81±0.11a	57.32±0.25a	***

Table S2. Chemical parameters determined during steel aging time (month): 1, 3 and 5.

→continued

Parameters	Vinification									
	1 month of steel aging				S.S.	3 month of steel aging				S.S.
	T1	T2	C1	C2		T1	T2	C1	C2	
pH	3.39±0.01b	3.39±0.02b	3.41±0.00b	3.46±0.01a	***	3.40±0.01b	3.39±0.01b	3.43±0.00a	3.44±0.02a	*
TA	5.40±0.01a	5.40±0.08a	5.49±0.03a	5.45±0.10a	n.s.	5.38±0.09a	5.39±0.02a	5.45±0.04a	5.46±0.10a	n.s.
VA	0.30±0.00c	0.30±0.00c	0.35±0.01b	0.43±0.00a	***	0.33±0.00b	0.34±0.01b	0.36±0.00a	0.36±0.00a	*
Rs	3.00±0.02b	2.90±0.01c	2.51±0.00d	3.36±0.02a	***	1.25±0.02b	1.19±0.02c	1.15±0.03c	1.47±0.01a	***
Glu	1.62±0.02a	0.47±0.00c	0.43±0.00d	1.47±0.02b	***	0.31±0.01b	0.30±0.00b	0.35±0.02a	0.21±0.01c	***
Fru	1.38±0.00d	2.43±0.01a	2.08±0.00b	1.89±0.00c	***	0.94±0.01b	0.89±0.02c	0.80±0.01b	1.26±0.00a	***
Eth	11.35±0.08a	11.43±0.13a	11.36±0.08a	11.35±0.07a	n.s.	11.35±0.07a	11.43±0.10a	11.36±0.00a	11.35±0.09a	n.s.
Gly	5.68±0.08bc	5.61±0.07c	5.82±0.03b	6.48±0.08a	***	5.67±0.06bc	5.60±0.09c	5.82±0.00b	6.49±0.08a	***
MA	1.35±0.02a	1.33±0.02a	1.32±0.03ab	1.27±0.01b	**	1.34±0.01a	1.32±0.01a	1.33±0.01a	1.27±0.00b	*
LA	0.00±0.00a	0.00±0.00a	0.00±0.00a	0.00±0.00a	n.s.	0.00±0.00a	0.00±0.00a	0.00±0.00a	0.00±0.00a	n.s.
Amm. N	n.d.	n.d.	n.d.	n.d.	n.d.	n.d.	n.d.	n.d.	n.d.	n.d.
Alpha-AN	n.d.	n.d.	n.d.	n.d.	n.d.	n.d.	n.d.	n.d.	n.d.	n.d.

Result indicate mean value ± standard deviation of three determinations. Data in the same line followed by the same letter are not significantly different according to Tukey's test.
Abbreviations: TA, total titratable acidity (tartaric acid g/l); VA, volatile acidity (acetic acid g/L); RS, reducing sugar (g/L); Glu, glucose (g/L); Fru, fructose (g/L); Eth, ethanol (% v/v); Gly, glycerol (g/L); MA, malic acid (g/L); LA, lactic acid (g/L); Amm. N, ammoniacal nitrogen (mg/L); Alpha-AN, alpha-amino nitrogen (mg/ L); n.d., not determined. P value: *, $P < 0.05$; **, $P < 0.01$; ***, $P < 0.001$; n.s., not significant.

Parameters	Vinification				
	5 month of steel aging				S.S.
	T1	T2	C1	C2	
pH	3.38±0.01b	3.38±0.02b	3.41±0.00a	3.43±0.00a	***
TA	5.35±0.06a	5.38±0.05a	5.38±0.10a	5.42±0.05a	n.s.
VA	0.34±0.00c	0.36±0.01b	0.35±0.00b	0.38±0.00a	***
Rs	0.61±0.00a	0.50±0.03b	0.28±0.01d	0.31±0.01c	***
Glu	0.20±0.00a	0.20±0.01a	0.12±0.01b	0.15±0.01b	***
Fru	0.41±0.00a	0.30±0.02b	0.16±0.00c	0.16±0.00c	***
Eth	11.35±0.17a	11.43±0.02a	11.36±0.06a	11.35±0.08a	n.s.
Gly	5.64±0.08bc	5.59±0.09c	5.80±0.01b	6.47±0.06a	***
MA	1.32±0.02a	1.31±0.02ab	1.30±0.00ab	1.27±0.02b	**
LA	0.00±0.00a	0.00±0.00a	0.00±0.00a	0.00±0.00a	n.s.
Amm. N	n.d.	n.d.	n.d.	n.d.	n.d.
Alpha-AN	n.d.	n.d.	n.d.	n.d.	n.d.
